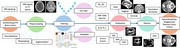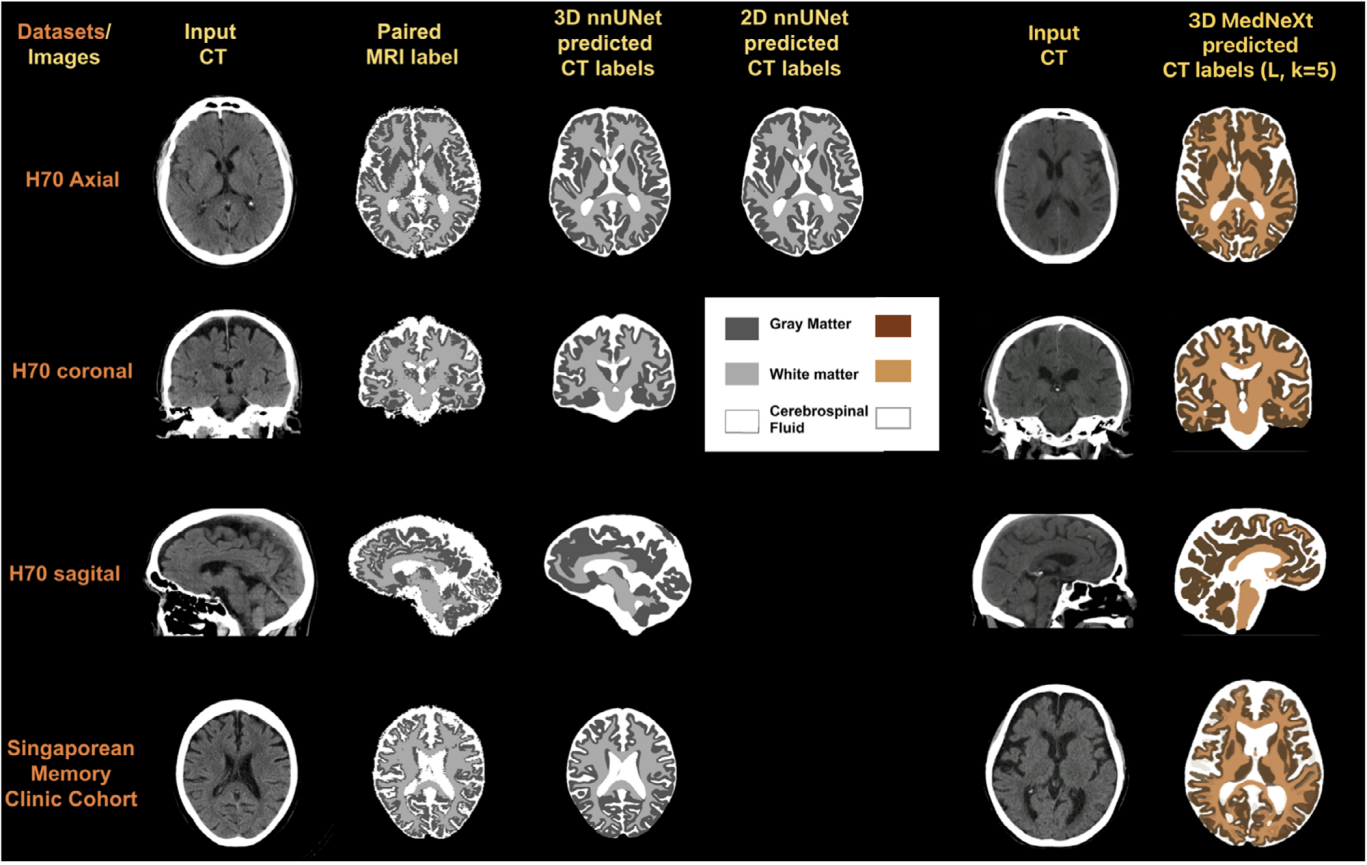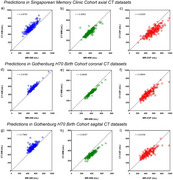# Automated Brain Tissue Segmentation on CT guided by MRI: Advancing AI‐based Neuroimaging for Dementia

**DOI:** 10.1002/alz70856_104843

**Published:** 2026-01-10

**Authors:** Vidya Somashekarappa, Meera Srikrishna, Silke Kern, Joyce R Chong, Eric Westman, Christopher Chen, Ingmar Skoog, Jakob Seidlitz, Michael Schöll

**Affiliations:** ^1^ Institute of Physiology and Neuroscience, University of Gothenburg, Gothenburg, Gothenburg, Sweden; ^2^ Wallenberg Centre for Molecular and Translational Medicine, University of Gothenburg, Gothenburg, Gothenburg, Sweden; ^3^ Wallenberg Centre for Molecular and Translational Medicine, University of Gothenburg, Gothenburg, Sweden; ^4^ Institute of Neuroscience and Physiology, Sahlgrenska Academy, University of Gothenburg, Gothenburg, Sweden; ^5^ Neuropsychiatric Epidemiology, Institute of Neuroscience and Physiology, Sahlgrenska Academy, Centre for Ageing and Health (AGECAP) at the University of Gothenburg, Gothenburg, Sweden; ^6^ Department of Psychiatry and Neurochemistry, Institute of Neuroscience and Physiology, The Sahlgrenska Academy at the University of Gothenburg, Mölndal, Vastra Gotaland, Sweden; ^7^ Sahlgrenska University Hospital, Mölndal, Gothenburg, Sweden; ^8^ Memory Aging and Cognition Center, National University Health System, Singapore, Singapore; ^9^ Memory, Ageing, and Cognition Centre (MACC), Department of Pharmacology, Yong Loo Lin School of Medicine, National University of Singapore, Singapore, Singapore; ^10^ Division of Clinical Geriatrics, Center for Alzheimer Research, Department of Neurobiology, Care Sciences and Society, Karolinska Institutet, Stockholm, Sweden; ^11^ Memory, Ageing and Cognition Centre, National University Health System, Singapore, Singapore; ^12^ Department of Pharmacology, National University of Singapore, Singapore, Singapore; ^13^ Department of Child and Adolescent Psychiatry and Behavioral Science, The Children's Hospital of Philadelphia, Philadelphia, PA, USA; ^14^ Penn/CHOP Lifespan Brain Institute, University of Pennsylvania, Philadelphia, PA, USA; ^15^ Department of Psychiatry and Neurochemistry, Institute of Physiology and Neuroscience, University of Gothenburg, Gothenburg, Västra Götalands län, Sweden; ^16^ Department of Neurodegenerative Disease, UCL Institute of Neurology, London, United Kingdom; ^17^ Department of Neuropsychiatry, Sahlgrenska University Hospital, Gothenburg, Gothenburg, Sweden; ^18^ University of Gothenburg, Gothenburg, Västra Götalands län, Sweden

## Abstract

**Background:**

Brain tissue segmentation is vital in Alzheimer's and dementia research for creating detailed neuroanatomical maps, diagnosing early‐stage neurodegeneration, and guiding interventions. Although MRI remains the standard approach for its superior soft‐tissue contrast, CT is a more accessible imaging modality in acute and resource‐constrained settings.

**Method:**

This study utilized paired CT‐MRI datasets from the Gothenburg H70 Birth Cohort (*N* = 733) and the Memory Clinic Cohort of the National University Hospital, Singapore (NUS Dementia Cohort, *N* = 210) to train and evaluate advanced segmentation models—**nnUNet** (2D & 3D models for 300‐1000 epochs) and **MedNeXt** (3D‐ Small, Base, Medium and Large models for 3x3x3 & 5x5x5 kernels). MRI‐derived labels were employed to guide CT segmentation, allowing accurate delineation of brain tissue segmentation (Gray Matter: GM, White Matter: WM and Cerebrospinal Fluid: CSF). Evaluation was conducted on all axial datasets for all variations of the models and for coronal & sagittal orientations the best performing models were utilized for inference.

**Result:**

The 3D nnU‐Net achieved average Dice Similarity Coefficients (DSCs) of 0.82, 0.72, and 0.76 for axial, coronal, and sagittal orientations, respectively, while MedNeXt demonstrated slightly superior performance with DSCs of 0.83, 0.73, and 0.78. MedNeXt also exhibited improved volumetric similarity in axial datasets, with scores ranging from 0.842 (CSF, sagittal) to 0.992 (WM, axial). When applied to dementia cohorts, MedNeXt achieved higher generalizability with an average DSC and volumetric similarity of 0.73 and 0.912, compared to 0.70 and 0.854 for nnU‐Net. Extended training (1000 epochs) enhanced nnU‐Net's performance, yet MedNeXt displayed superior scalability, handling larger kernel sizes and multi‐modal imaging scenarios. However, significantly longer training times of up to 288 hours was required for the largest model.

**Conclusion:**

Automated CT brain segmentation guided by MRI‐derived labels demonstrates clinically acceptable segmentation performance on untrained dementia cohort. nnU‐Net is more resource‐efficient and suitable for limited‐resource settings, while MedNeXt has higher accuracy excelling in multi‐orientation and multi‐modal datasets. These findings validate the feasibility of using CT imaging with advanced segmentation frameworks to develop accessible neuroimaging tools for Alzheimer's and dementia research, addressing diagnostic challenges across diverse clinical contexts.